# Optimization of an Inductive Displacement Transducer

**DOI:** 10.3390/s23198152

**Published:** 2023-09-28

**Authors:** Bogdan Mociran, Marian Gliga

**Affiliations:** Faculty of Electrical Engineering, Technical University of Cluj-Napoca, 28 Memorandumului Street, 400114 Cluj-Napoca, Romania; marian.gliga@ethm.utcluj.ro

**Keywords:** interior inductive displacement transducer, linearization, optimization, parameter improvement, sensor performance

## Abstract

This paper presents the optimization of an inductive displacement transducer or linear variable differential transformer (LVDT). The method integrates design software (SolidWorks 2023), simulation tools (COMSOL Multiphysics), and MATLAB. The optimization phase utilizes the non-dominated sorting genetic algorithm (NSGA)-II and -III to fine-tune the geometry configuration by adjusting six inner parameters corresponding to the dimension of the interior components of the LVDT, thus aiming to improve the overall performance of the device. The outcomes of this study reveal a significant achievement in LVDT enhancement. By employing the proposed methodology, the operational range of the LVDT was effectively doubled, extending it from its initial 8 (mm) to 16 (mm). This expansion in the operational range was achieved without compromising measurement accuracy, as all error values for the working range of 0–16 (mm) (NSGA-II with a maximum final relative error of 2.22% and NSGA-III with 2.44%) remained below the imposed 3% limit. This research introduces a new concept in LVDT optimization, capitalizing on the combined power of NSGA-II and NSGA-III algorithms. The integration of these advanced algorithms, along with the interconnection between design, simulation, and programming tools, distinguishes this work from conventional approaches. This study fulfilled its initial objectives and generated quantifiable results. It introduced novel internal configurations that substantially improved the LVDT’s performance. These achievements underscore the validity and potential of the proposed methodology in advancing LVDT technology, with promising implications for a wide range of engineering applications.

## 1. Introduction

Linear variable differential transformers (LVDTs) are currently vastly exploited in engineering applications as well as in adjacent fields. They are generally used for their effective results in measuring distance, position, pressure, or force [1]. The electrical equipment that is a part of this device is considered to be robust and reliable, and it provides high measurement sensitivity [2]. A standard LVDT device consists of three major components: the shell, the mobile magnetic core, and the coils (that wrap around the magnetic core). The secondary coils are connected in series opposition, one facing the other, and between the two secondary coils, the primary coil is placed. The mobile magnetic core slides inside the LVDT on a straight line. The primary feature of an LVDT device lies in its ability to exhibit a linear response to displacement. The constraints encountered during the optimization of such a device involve maximizing linearity while considering a predefined tolerance value, as demonstrated in [3]. Moreover, the benefits of introducing finite-element modeling in the development of LVDTs have been proven in [4]. A large portion of the available studies focus on utilizing neural networks to assist in compensating for the nonlinear data output that presents itself when an LVDT is being used at the extremity of the work scale. The results obtained in [5] indicate that utilizing a radial basis function neural network for nonlinearity compensation in LVDT output is an effective method and holds significant implications for accurate displacement measurements. Using a functional link artificial neural network (FLANN) to address LVDT nonlinear compensation, and showcasing its efficacy through comparative simulations with experimental data, has been accomplished, and additionally, the authors of that previous work provide the detailed algorithm and setup used in their study [6]. The authors continued their work based on the same idea by using a single-layer FLANN in [7] and expanding the testing to two different LVDTs. A new two-stage FLANN was used for enhancing the linearity of an LVDT [8]; they applied first a lower-order FLANN for rough approximation of the linearity, and after that, a high-order FLANN was used to complete the improvement. An approach to expanding the work field where LVDT can operate, based on an analog lookup table, can be found here [1]. Another studied approach is based on the attempt to compensate for the nonlinearity of the output in the secondary coils by applying different suitable mathematical nonlinear functions on the initial signal generated by an LVDT [9].

The authors in [10] present a circuit technique using hyperbolic sine functions to linearly extend the measuring range of a commercial LVDT, effectively compensating for its inherent nonlinearities without requiring digital components, resulting in a significantly increased linear range.

The calculation of eddy current losses in LVDTs is addressed in [11]. The authors introduce a new methodology to tackle this problem, particularly for LVDTs with an open-type core, with the aim of improving the design to obtain greater measurement accuracy. Former research attempts aimed at improving the reactivity and robustness of the LVDT, focusing on the dissimilarities between different materials from which the device was built (mainly for the magnetic core that was forged from soft magnetic material, [12]). Taking the idea of testing different materials into account [13], the authors from the cited source propose a less common analysis in which the mobile magnetic core is no longer built out of solid magnetic material but from a liquid one, i.e., ferrofluid, and recommend an analysis on the differences in linearization depending on the material and its temperature.

By predicting the fatigued strength of the materials and analyzing their distribution, more suitable materials can be selected and used for LVDT components to avoid premature failures [14]. Consequently, this approach could lead to improved performance and reliability of LVDT position sensors [15]. For casting certain components of an LVDT, such as the outer casing, material fatigue may occur in some cases. Studying the prediction of material fatigue and considering this phenomenon can be beneficial in LVDT design [16].

In a previous work [17], the authors made a significant contribution to the interconnection process by innovating the framework. This framework enables the seamless integration and communication between various design programs (e.g., computer-aided design CAD software SolidWorks 2023), simulation tools (e.g., FEA software COMSOL Multiphysics 4.3), and optimization methods (e.g., genetic algorithms). By compiling multiple programs and algorithms, the new designed framework capacitates engineers to benefit from the strengths of these tools, thus enhancing the efficiency and effectiveness of the process in its entirety.

In this paper, our objective is to address the issue of LVDT linearization using two optimization methods: the NSGA-II and the NSGA-III algorithms, which are integrated into the system described in [17]. Our contribution lies in applying these methods to enhance the performance of the LVDT by increasing its working distance in comparison to the initial analyzed model in [3], through a simple yet efficient approach. By modifying the internal geometrical configuration (while maintaining the external dimensions unchanged), we achieved notable results regarding the above-mentioned goals. It is important to clarify that the present work focuses solely on simulation and optimization and does not involve the implementation or testing of practical applications. This will be addressed in a future study, where we will explore the applicability and performance of our models in a real-world context.

## 2. Materials and Methods

The method proposed in this paper is in fact a component of a wider project for development and optimization, aimed at assisting the creation of a large palette of engineering equipment. Considering the significantly increasing need for theoretical research results to be implemented in industries and the fact that the geometries that are applied in the lines of work are 3D and complex shapes, it was decided that the testing be carried out for LVDT in this way. By doing so, the axisymmetric feature of the model was bypassed.

The LVDT study was done by using the module Magnetic Fields interface for 3D geometry in combination with the frequency domain found in the COMSOL Multiphysics tool for calculating the magnetic field and the induced current, as well as the induced voltage in the coils. In this case, Maxwell’s equations are resolved with the assistance of the magnetic vector potential A with the components Ax, Ay, and Az, respectively.

In order to reach the aimed purpose, i.e., to expand the working area of the proposed LVDT, the minimization of the objective function Equation (1) is needed. Taking into account the proposed constraints of the design variables from Equation (2), one can notice the intention to only adjust the dimensions of the internal components without any impact on their shape.

(1) $$f\left(p\right)=\frac{1}{2}\overset{s}{\underset{n=1}{\sum }}({u_{n}}^{c}-{u_{n}}^{\mathrm{fix}}{)}^{2}$$

where *s*—represents the number of predefined positions of the magnetic core (*s* = 10 points), in which the induced voltage is calculated; u_n_^fix^—the predefined values of the induced voltage desired to be reached once the optimization is done for each different position “n”, where the moving magnetic core is found; u_n_^c^—the values of the induced voltage in the secondary coils, calculated by the COMSOL Multiphysics 4.3 software for each “n”. In addition,

(2) $$1\leq p_{1}\leq 24.50\;(\mathrm{mm});\;1\leq p_{2}\leq 28\;(\mathrm{mm});\;1\leq p_{3}\leq 5\;(\mathrm{mm});\;1\leq p_{4}\leq 5\;(\mathrm{mm});\;1\leq p_{5}\leq 6\;(\mathrm{mm});\;6\leq p_{6}\leq 32\;(\mathrm{mm})$$

where p_1_, p_2_, p_3_, p_4_, p_5_, p_6_—represent the optimization parameters.

Equation (3) represents the initial design parameter vectors, where each value is identical with the geometrical dimension (in millimeters):

p_0_ = {15, 20, 2.25, 4.5, 4.5, 26.50}^T^ (mm) (3)

The materials used in setting up this model are the standard ones for air (for the internal space between the components of the LVDT) and copper (for the coils) found in the COMSOL Multiphysics 4.3 software, while the material used for the magnetic core was aligned to the following parameters for magnetic losses: relative permeability—the real part with the value of 1200 and the imaginary part with the value of 100.

**Figure 1:**
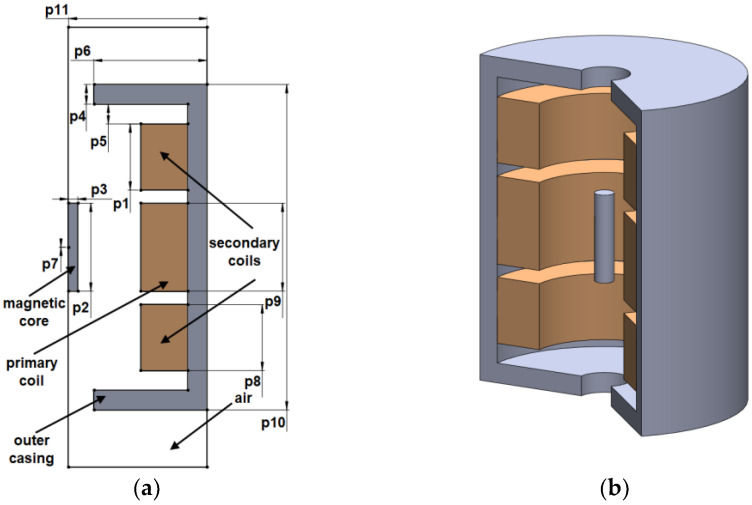
The configuration of the initial model in 2D (**a**) and 3D, respectively (**b**).

As in [3], the chosen device operates at a frequency of 1 (kHz), having the excitation current applied to the primary coil. All three coils depicted in brown in Figure 1 (one primary and two secondary coils) have 100 turns with the cross-section of the conductor of 1 × 10^−6^ (m^2^). The dimensions of the geometry used are detailed in Table 1 together with the value of the objective function for the initial configuration of the test model. 

NSGA-II is an advanced version of the original NSGA algorithm, which integrates a genetic algorithm framework with a non-dominated sorting and crowding distance mechanism. This combined approach efficiently guides the search process towards the Pareto front, where the optimal solutions reside. These solutions are characterized by the property that no individual solution can be enhanced in one objective without compromising its performance in another. NSGA-II is a multi-objective optimization algorithm that generates an initial population of candidate solutions and applies genetic operators, such as crossover and mutation, to create offspring. It then employs non-dominated sorting to assign ranks based on dominance relationships and utilizes crowding distance to maintain diversity within each rank. The algorithm iteratively evolves the population, favoring solutions along the Pareto front, until a termination condition is met [18].

NSGA-III is an advanced multi-objective optimization algorithm, developed upon NSGA-II. It exploits a genetic algorithm framework, non-dominated sorting, with crowding distance. NSGA-III improves convergence and diversity in high-dimensional objective spaces by introducing reference points. The algorithm iteratively generates candidate solutions, applies genetic operators, and evolves the population towards a diverse set of Pareto-optimal solutions [19].

As the authors of the work [3] demonstrate, the objective function shows a series of local minima, and so the use of stochastic tools such as NSGA-II and -III is preferred to tackle the problem as opposed to the deterministic algorithms.

By using NSGA-II and NSGA-III, we aim to observe the results in different conditions, aside from the known ones. Also, we aim to compare if there are different optimal configurations generated for the same initial geometry of the LVDT, not to mathematically test the difference between the two algorithms, which is already known in specialized literature.

**Figure 2:**
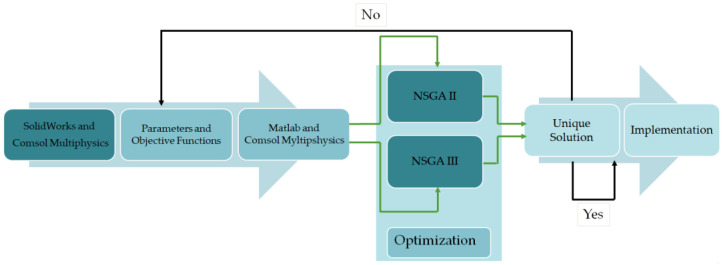
The block diagram of the proposed optimization system.

The proposed algorithm for the improvement of the LVDT studied in this paper is split into two optimization directions, one based on the NSGA-II algorithm and the other on NSGA-III; see the block diagram shown in Figure 2. As it can be observed in the figure, the process is a cyclical one, meaning that if the obtained solution is not satisfactory, then the entire process can be restarted without the need of redoing the whole setup, simply by adjusting the input that the two algorithms require to run (population size, the number of generation). It is well known that a large population size and a high number of generations that are used by the algorithm would generate better results but with the inconvenience of a longer processing time.

We need to consider that these algorithms are multi-objective algorithms for optimization; however, in the studied case, we only have one objective function that requires improvement. To overcome this obstacle, a logical trick was used, meaning that the initial objective function values were duplicated to obtain two optimization objective functions with identical values. Moreover, these functions, in this context, do not influence each other while the parameters are subjected to the optimization process change.

### 2.1. The First Approach

**Figure 3:**
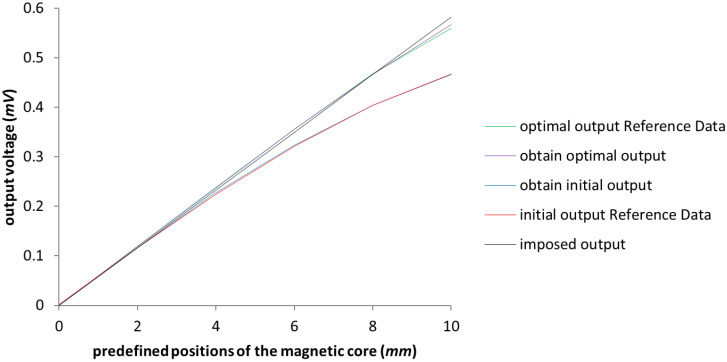
The graphical representation of the calibration between initial model and test model regarding the output voltage. The green and red lines represent the Reference Data results outlined in [3].

**Figure 4:**
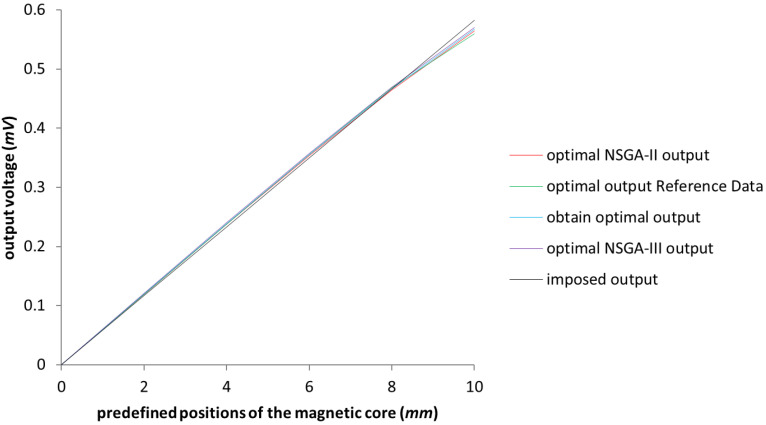
Graphical representation of the optimization. Comparison between the initial and the test models. The green line represents the Reference Data results outlined in [3].

The model from [3] was replicated in order to have it as an initial consistent set of data. The testing was done with the un-optimized model in the first instance. It can be observed in Figure 3 that the starting values coincide with the ones presented in [3], represented in the chart below by the red and blue lines, which are almost perfectly overlapping, thus demonstrating the accuracy of the replicated initial test model. To ensure that not only the initial model was calibrated, a simulation was proposed with the values of the obtained optimization parameters achieved in [3], and the results, as it can be seen in Figure 3 represented in green and purple, are rightly calibrated as well. The testing of the new optimization system was ensured through a comparison between the obtained results and the ones exemplified in [3]. For the optimization of the LVDT test model, the algorithms used were NSGA-II and NSGA-III, in which only the design variables were taken into account, i.e., the length of the secondary coil and the length of the magnetic core, in order to replicate the workings done in [3]. The obtained results reflect the initial expectations and can be seen in Figure 4, where the values of the output voltage are almost identical; the slight differences between these four sets of data are negligible.

**Figure 5:**
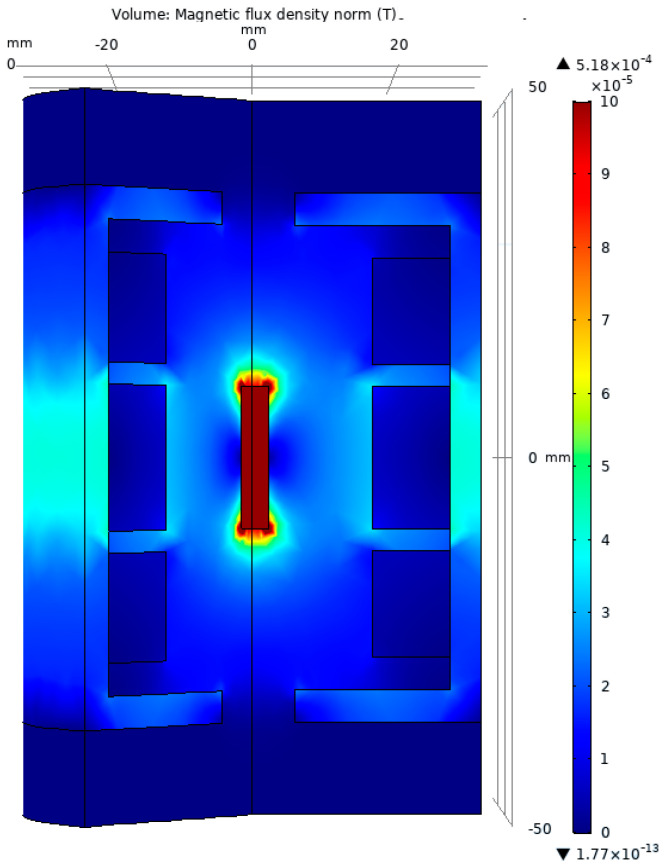
The graphical representation of the magnetic flux density for the test model.

The magnetic flux density for the test model is presented in Figure 5. One can observe that the mobile magnetic core is situated at the center of the LVDT, corresponding to the standard initial position 0 (mm).

The 3D representation of the test model was chosen in such a way as to allow the viewing of the interior components of the LVDT. In this image, we can see, as expected, the concentration of the magnetic field on the mobile core, which is built from soft magnetic material.

### 2.2. The Second Approach—Extending the Operational Range from 0 to 18 (mm)

**Figure 6:**
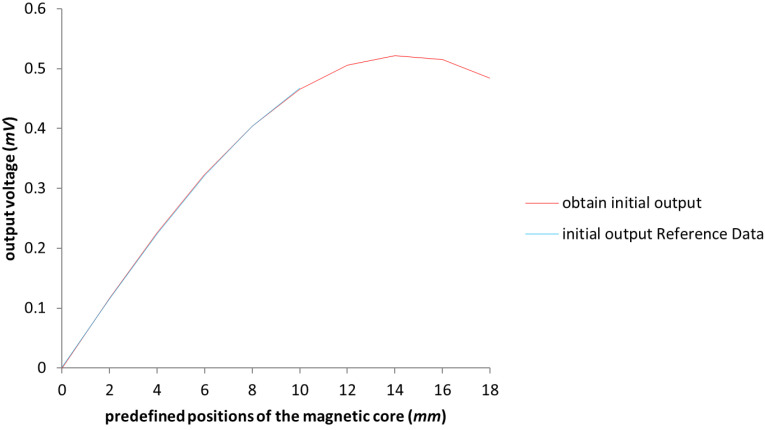
The graphical representation of the values of the LVDT with the extension of the work area up to 18 (mm). The blue line represents the Reference Data results outlined in [3].

In this case, all six parameters, i.e., p₁–p₆, were considered in the attempt to improve the test model. The obtained results for the configuration presented in Table 1 with the expansion of the moving distance of the mobile magnetic core up to the value of 18 (mm) are shown in Figure 6. We can see how the lines overlap (the blue line representing the obtained response of the initial model and the red one representing the response of the test model). This demonstrates the accuracy of the test model. It can be noticed that the result marked in red has an emphasized deformity (nonlinear response) that appears once the magnetic core moves over 4 (mm), up to which the LVDT was acting within the imposed functioning requirements. Once the core passes the distance of 8 (mm), the response is even more deformed (showing a parabola shape to the output voltage) in comparison to the results of the objective of having a linear output with the movement.

## 3. Results

### 3.1. NSGA-II Optimization

After setting the test model up by building the geometrical shape, assigning boundary conditions, and meshing the geometry needed for the FEM calculating method, the actual optimization via the NSGA-II algorithm was undertaken.

The geometry of the optimized model has the configuration exemplified in Table 2.

**Figure 7:**
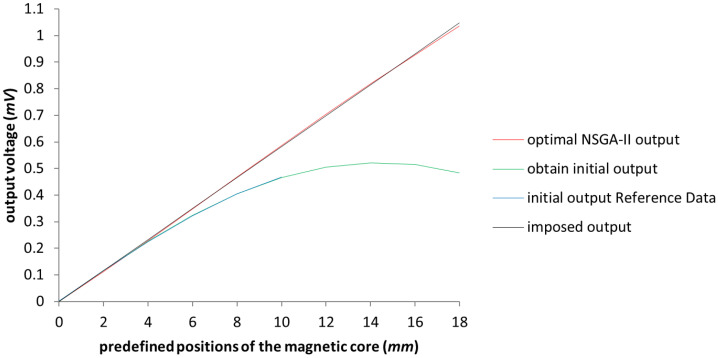
The graphical representation of the output voltage for the NSGA-II optimized model with the expansion of the linage up to 18 (mm). The blue line represents the Reference Data results outlined in [3].

**Figure 8:**
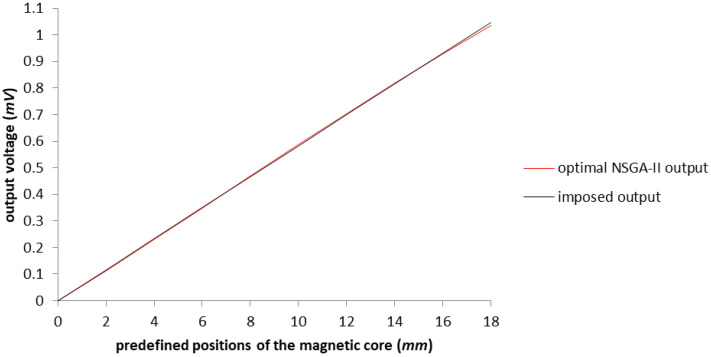
The graphical representation of the output voltage for optimized model with the NSGA-II algorithm in comparison with the imposed output.

In Figure 7, the improvement of the voltage output is represented in red near the perfect linearization imposed (represented by the black line). Expanding the working area up to 16 (mm), we reach a response that is below the maximum error tolerance. For a better understanding of the improvement, in Figure 8 we only have the imposed output and the obtained characteristics of the LVDT, after applying the NSGA-II algorithm upon the test model.

**Figure 9:**
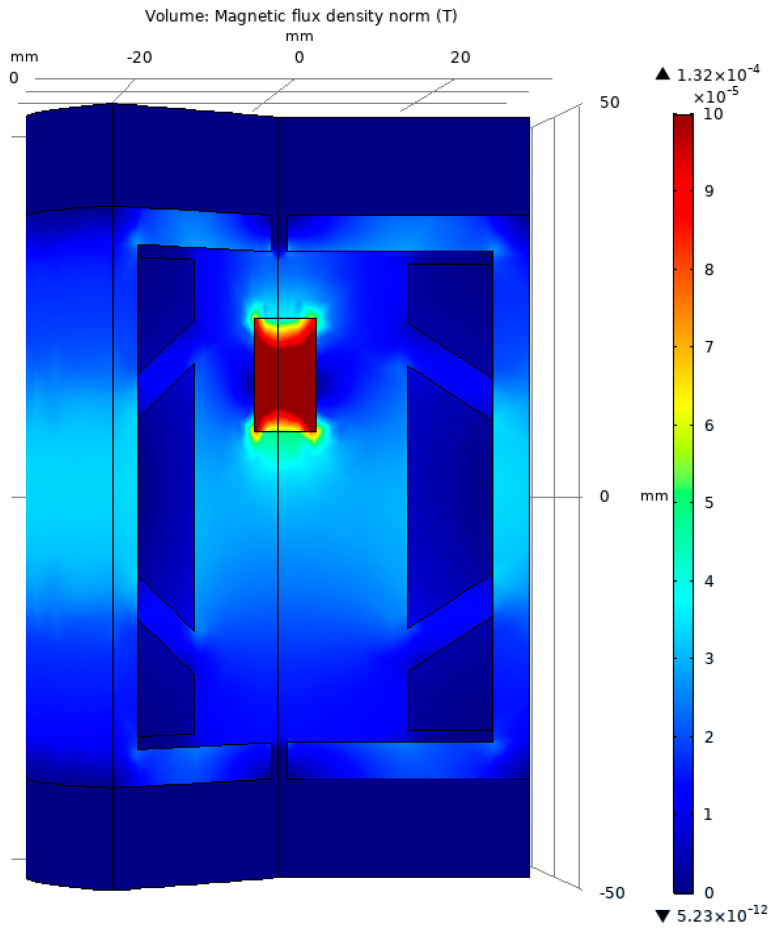
The graphical representation of the magnetic flux density for the test model optimized with the NSGA-II algorithm.

The results of the optimal configuration can also be observed in Figure 9, which gives a 3D representation of the magnetic flux density. The geometry of the primary and secondary coils has been altered, decreasing the size of the side of the secondary coils exposed to the magnetic core and increasing the primary coil at the same rate.

### 3.2. NSGA-III Optimization

For this case study, the geometry of this final optimization model has the design variables outlined in Table 3.

**Figure 10:**
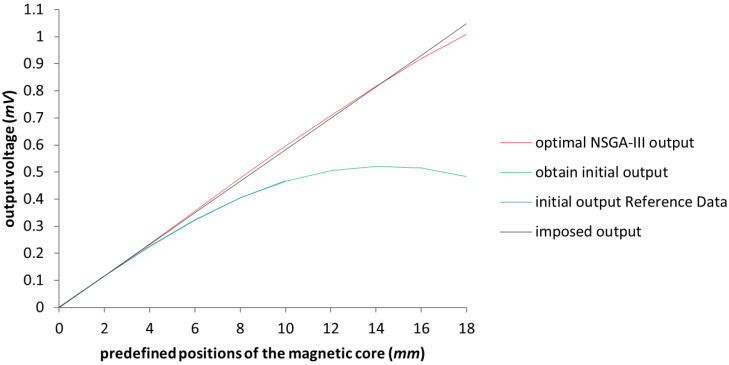
The graphical representation of the output voltage NSGA-III optimized model with the expansion of the work area up to 18 (mm). The blue line represents the Reference Data results outlined in [3].

**Figure 11:**
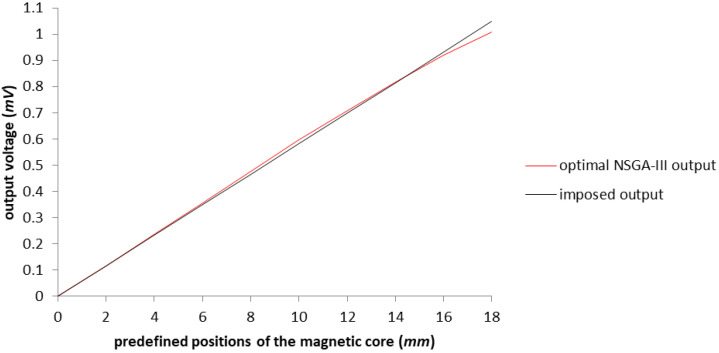
The graphical representation of the output voltage for the optimized model with the NSGA-III algorithm in comparison with the imposed output.

Again, represented in red in Figure 10, we have the voltage output for the configuration obtained with NSGA-III, which demonstrates the linearization from 0 to 16 (mm), within the value of the maximum error tolerated. Figure 11 gives a more simple graphical representation of the imposed output and the results of the best configuration achieved after NSGA-III was applied to the test model.

**Figure 12:**
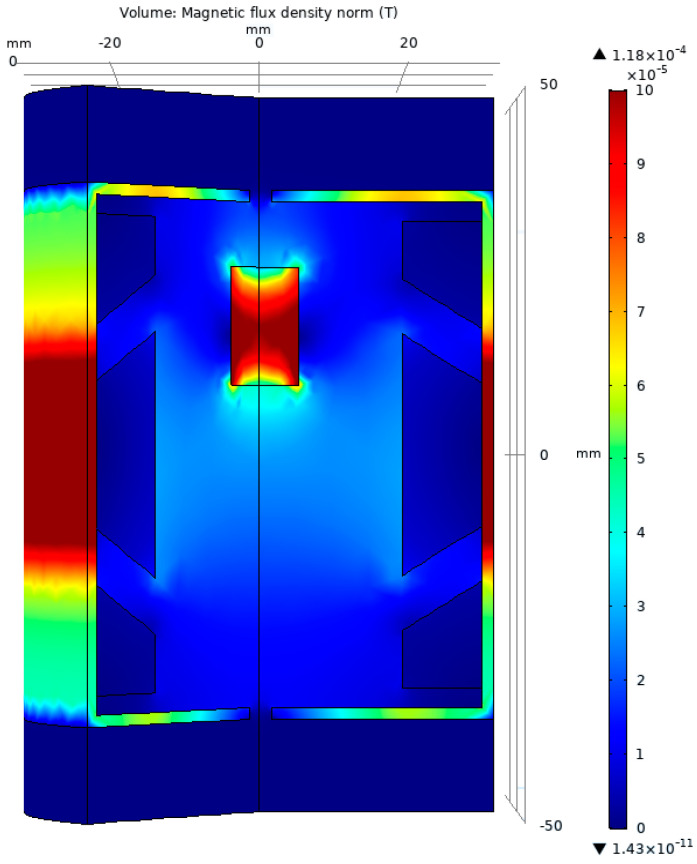
The graphical representation of the magnetic flux density for the test model optimized with the NSGA-III algorithm.

Figure 12 gives a 3D representation of the distribution of the magnetic flux density upon the geometry generated with the NSGA-III algorithm. Moreover, the coils are altered in size; the secondary coils exposed to the magnetic core decrease, while the primary coil increases, at the same rate.

## 4. Discussion

### 4.1. The Discussion Based on the NSGA-II Optimization

The necessary calculation time spent for this optimization attempt was 26 h and 43 min, having an initial mesh of 17,923 tetrahedrons, in comparison to the geometry of the optimized model that contains a number of 20,190 tetrahedrons.

The convergence of the objective function has required a run with 291 steps in order to be finalized at a population size of 50 individuals with 5 generations. The objective function decreased in this case with a magnitude of 100, in comparison to the starting value found in Table 1.

**Figure 13:**
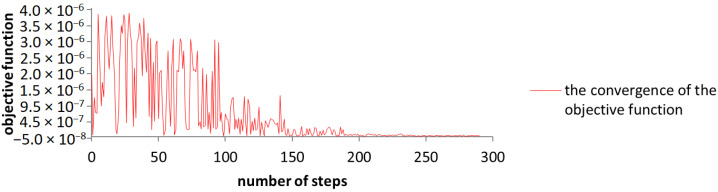
The graphical representation of the convergence of the objective function while using the NSGA-II algorithm.

The graphical representation of the convergence of the objective function (Figure 13) shows that the algorithm manages to find the best solution after approximately 150 steps, where the values stabilize at the optimal value.

**Figure 14:**
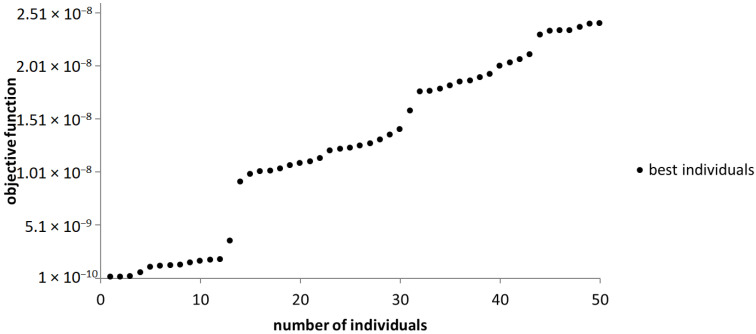
The graphical representation of the best individuals that resulted from the use of the NSGA-II algorithm.

Out of the 291 steps accomplished, the algorithm generates the best 50 individuals out of which the optimal version is selected. As pointed out in Figure 14, the unique solution is chosen to be the set of the design variables corresponding to the individual near the origin of the axis.

### 4.2. The Discussion Based on the NSGA-III Optimization

For this situation, the necessary calculation time was 26 h and 50 min, with an initial mesh of 17,923 tetrahedrons, in comparison to the geometry of this final optimization model that contains a number of 27,786 tetrahedrons.

The convergence of the objective function has lasted for 306 steps in order to be accomplished at a population size of 50 individuals with five generations. In this case, the decrease in the objective function has a magnitude of only 10 in comparison to the test model. A desired optimization is achieved, within the required parameters, in this simulation as well.

**Figure 15:**
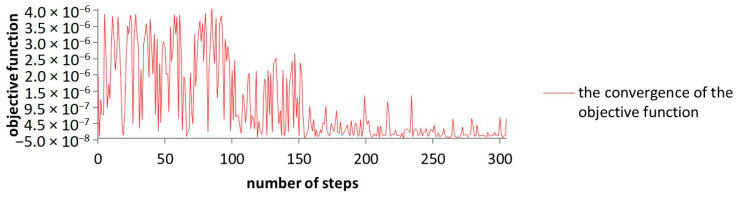
The graphical representation of the convergence of the objective function while using the NSGA-III algorithm.

In Figure 15, a less smooth convergence can be seen; however, after approximately 150 steps, an optimal configuration is reached.

**Figure 16:**
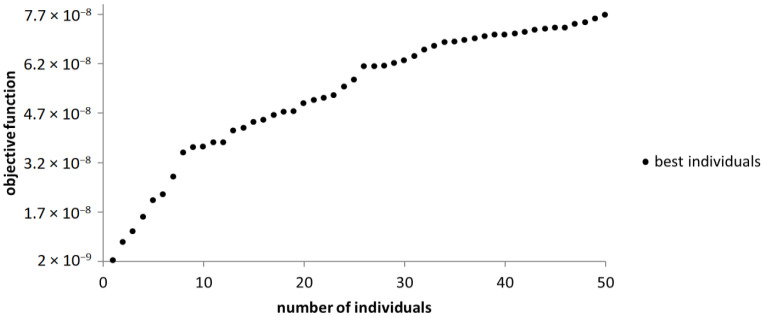
The graphical representation of the best individuals that resulted from the use of the NSGA-III algorithm.

Also, in this case, NSGA-III returns the best 50 individuals, from which the optimal version is selected. The chosen version represents the individual found adjacent to the origin of the axis (Figure 16).

### 4.3. Results Comparison

**Figure 17:**
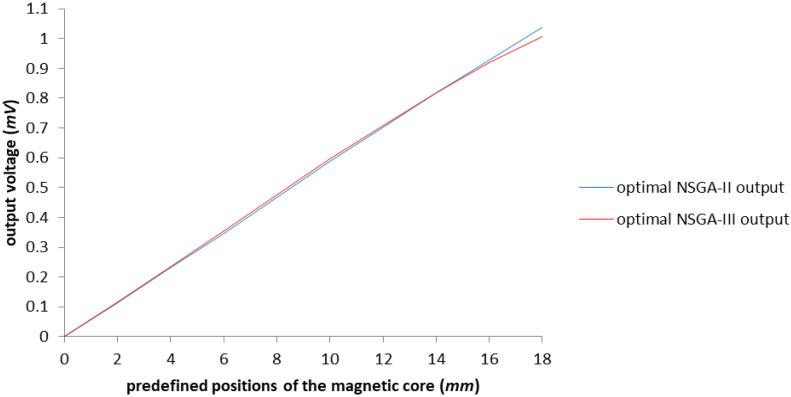
The graphical representation of the comparison between optimization results made with NSGA-II and NSGA-III.

Analyzing the data obtained from completing the two case studies, we can observe a more than satisfying improvement in each situation. The NSGA-III algorithm generates lesser results than the NSGA-II algorithm in the sense that the former manages to give the results in approximately the same amount of time as the latter, the difference being only 7 min; however, it manages to find a new configuration in which the casing is much thinner (three times thinner). The rest of the parameters are close to the same values. Considering the difference between the models, we can conclude that the results are similar, as exemplified in Figure 17, where the results are overlapping, proving that there is not a single optimal configuration where there is a high number of parameters to consider.

**Figure 18:**
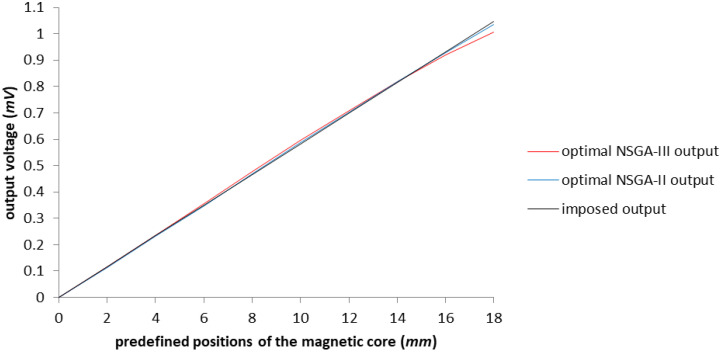
The graphical representation of the comparison between optimization results obtained with NSGA-II and NSGA-III regarding the imposed output.

Figure 18 sustains the idea that the results are satisfactory in comparison with the imposed output, despite the two different geometries achieved after the optimization.

For a comprehensive understanding of the differences between the two models, we can refer to the numerical data presented in Table 4 and Table 5. The “No data” cells in these tables are a result of the lack of research conducted in [3] regarding the movement of the magnetic core from 12 (mm) to 18 (mm). This improvement was proposed and achieved by the present study.

**Figure 19:**
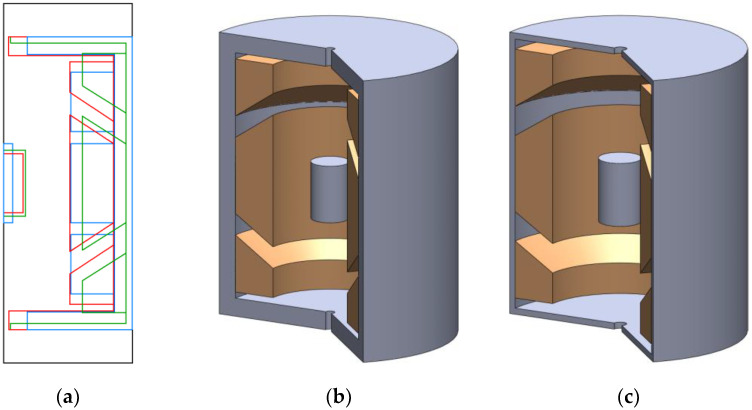
(**a**) A two-dimensional section of the model’s details compared to the simulation model configurations (blue), the model optimized with NSGA-II (red), and the model optimized with NSGA-III (green). The configurations of the model optimized with NSGA-II in 3D (**b**) and the model optimized with NSGA-III in 3D (**c**).

Figure 19a displays a two-dimensional representation of the model’s intricate features, and it highlights the variations between the different optimization approaches. The blue configuration represents the baseline simulation model, while the red and green configurations represent the outcomes of the optimizations achieved through NSGA-II and NSGA-III, respectively. Moving on to subfigures b and c, we witness the model configurations in a three-dimensional view. Subfigure b illustrates the model optimized using NSGA-II, providing a more comprehensive perspective of how the optimization affects the overall structure. On the other hand, subfigure c exhibits the model optimized using NSGA-III, showcasing its distinctive features in 3D.

## 5. Conclusions

This study reveals a novel methodology that integrates design programs, simulation programs, and optimization algorithms, specifically NSGA-II and III. This approach aims to enhance the geometry of the LVDT to extend its operational capabilities from 8 (mm) to 16 (mm) while maintaining measurement errors below 3% compared to the specified characteristics. Our research yields significant findings and conclusions, demonstrating a doubling of the working range compared to the initial model obtained from a prior study [3], which was accurately replicated and calibrated.

The un-optimized model exhibits substantial deviations from the desired output. In the first two standardized positions of the magnetic core, the error falls within the acceptable tolerance range, with 0.297% for the 2 (mm) position and 2.89% for the 4 (mm) position. However, in the extreme position of 18 (mm), the error exceeds 116% relative to the specified value. The extension of the LVDT’s working range was achieved by optimizing six internal design variables corresponding to the inner components of the LVDT.

The efficacy of the selected optimization algorithms is evident in the results obtained. NSGA-II achieves a maximum final relative error of 2.22%, consistently maintaining errors below 3% across all ten standardized positions of the magnetic core. In comparison, NSGA-III yields slightly higher error at the 18 (mm) position, with the value of 4.02%. From a design configuration perspective, NSGA-III manages to attain a thinner casing configuration than NSGA-II, highlighting the absence of a singular solution when considering multiple optimization parameters. Overall, both optimization approaches successfully expand the LVDT’s operational range to 16 (mm) while preserving satisfactory linearity regarding the imposed outcome.

It is essential to acknowledge the limitations of this approach. Namely, NSGA-II and -III have proven to be time-consuming optimization algorithms, requiring approximately 26 h to complete. Future studies could explore additional optimization parameters and investigate the impact of different materials and operating conditions on LVDT performance. Furthermore, there is potential for achieving more efficient results by employing faster learning algorithms or advanced AI capabilities that can consider a broader range of functions and parameters, thereby facilitating a more comprehensive optimization process.

## Figures and Tables

**Table 1:** The values for the initial model.

Initial Model	Value
Length of the secondary coil—p1	15 (mm)
Length of the magnetic core—p2	20 (mm)
Width of the magnetic core—p3	2.25 (mm)
Thickness of the casing—p4	4.50 (mm)
Distance casing–secondary coil—p5	4.50 (mm)
Width of the casing—p6	26.50 (mm)
Standardized position of the magnetic core—p7	0, 2, 4, 6, 8, 10, 12, 14, 16, 18 (mm)
Length of the secondary coils that maintains the same values—p8	15 (mm)
Length of the primary coil that maintains the same values—p9	20 (mm)
Height of the LVDT that maintains the same values—p10	74 (mm)
Radius of the LVDT from the center of the magnetic core, at shell extremity, that maintains the same values—p11	32.50 (mm)
Value of the objective function—f(p_0_)	1.7334 × 10^−8^ (V^2^)

**Table 2:** The values for the optimized model with NSGA-II.

Parameter 2D Cross Section NSGA-II	Value
Length of the secondary coil	7.69 (mm)
Length of the magnetic core	14.87 (mm)
Width of the magnetic core	4.90 (mm)
Width of the casing	31.24 (mm)
Thickness of the casing	4.76 (mm)
Distance casing–secondary coil	1.61 (mm)
Standardized position of the magnetic core	0, 2, 4, 6, 8, 10, 12, 14, 16, 18 (mm)
Value of the objective function	2.37856 × 10^−10^ (V^2^)

**Table 3:** The values for the optimized model with NSGA-III.

Parameter 2D Cross Section NSGA-III	Value
Length of the secondary coil	8.06 (mm)
Length of the magnetic core	16.56 (mm)
Width of the magnetic core	5.53 (mm)
Width of the casing	30.76 (mm)
Thickness of the casing	1.62 (mm)
Distance casing–secondary coil	2.62 (mm)
Standardized position of the magnetic core	0, 2, 4, 6, 8, 10, 12, 14, 16, 18 (mm)
Value of the objective function	2.29 × 10^−9^ (V^2^)

**Table 4:** The imposed, the initial and the obtained optimized output voltages.

(mm)	0	2	4	6	8	10	12	14	16	18
Linear val (mV)	0	0.1164	0.2329	0.3493	0.4658	0.5822	0.6987	0.8151	0.9316	1.0479
Original output in [3] (mV)	0	0.1164	0.225	0.3215	0.404	0.4673	No data	No data	No data	No data
Optimal output in [3] (mV)	0	0.1183	0.2375	0.3555	0.4679	0.5596	No data	No data	No data	No data
Obtain val (mV)	0	0.01167	0.2264	0.3233	0.4041	0.4656	0.5056	0.5218	0.5152	0.4847
NSGA-II val (mV)	0	0.1139	0.2299	0.3467	0.4675	0.5866	0.7039	0.8188	0.9281	1.0361
NSGA-III val (mV)	0	0.1160	0.2354	0.3549	0.4770	0.5968	0.7089	0.8171	0.9191	1.0074
Initial rel. er (%)	0	0.2975	2.8902	8.0255	15.268	25.049	38.187	56.225	80.796	116.23
Rel.er. NSGA-II (%)	0	2.2208	1.2641	0.7458	0.3620	0.7536	0.7491	0.4509	0.3721	1.1442
Rel.er. NSGA-III (%)	0	0.3270	1.0766	1.5950	2.3495	2.4415	1.4515	0.2427	1.3585	4.0294

**Table 5:** The parameter initial values as well as the optimized ones.

Parameters	Initial Model	NSGA-II	NSGA-III
p₁ (mm)	15	7.69	8.06
p₂ (mm)	20	14.87	16.56
p₃ (mm)	2.25	4.90	5.53
p₄ (mm)	4.50	31.24	30.76
p₅ (mm)	4.50	4.76	1.62
p₆ (mm)	26.50	1.61	2.62

## Data Availability

Not applicable.
